# Clinical Records in Poor-Law Infirmaries: A Rejoinder

**Published:** 1915-01-30

**Authors:** 


### CLINICAL RECORDS IN POOR-LAW INFIRMARIES.

### A Rejoinder: By an Infirmary Medical Superintendent.

The writer of the article on "The Clinical Value of Poor-Law Infirmaries" in last week's Hospital does considerably less than justice to the amount of work in the way of clinical records carried on by the medical officers of these institutions. He suggests that in most cases no notes are kept at all, and states that when they are kept they are of the scantiest description. He even compares them unfavourably with the meagre records kept by the panel doctors. Here he makes a mistake which a very little investigation would have enabled him to avoid. He says that panel doctors are required to take notes and records of their diagnosis and treatment. This is not the case. The only records the panel doctor is required to keep are a bare statement of the name, age, sex, and occupation of the patient, the diagnosis and the dates of his attendances. No notes of the physical examination, of the progress of the case, or of the treatment are required from him.

Compare this with the requirements of the Local Government Board, as laid down in the Infirmary Orders and the Poor-Law Institutions Order. Under these Orders the medical officer is required to keep for every patient a case paper showing the name, age, sex, and occupation, and other particulars about the patient, the diagnosis, the history of the case before admission, the condition of the patient on admission, the diet and medical treatment ordered, a record of the progress of the case and the result, including the cause of death, if death occurs. With a somewhat wide acquaintance with the London and larger provincial infirmaries, I can testify from personal knowledge that these records are faithfully kept in most cases.

That the notes are not kept with the same wealth of detail as in the case of the London voluntary hospitals may be freely admitted. The reason is to be found, as the writer of the article points out, in the paucity of the medical staff. A Poor-Law infirmary with 500 beds will have a staff of three or, at the most, four medical officers. Compare this with the medical staff of a voluntary hospital of corresponding size, with its visiting staff of surgeons and physicians, its special departments and their staffs, its medical and surgical registers, its house physicians and house surgeons, and its army of clinical clerks, and the unfairness of comparing the clinical notes kept under the two systems is at once obvious. The notes kept at a Poor-Law infirmary are severely practical and to the point.

We may illustrate these points from the case selected by the writer of the article for adverse criticism. He states that a case of carbonic-acid gas poisoning was admitted to an infirmary, and that the notes of the case were valueless, because the only note made read: "Admitted as a supposed case of gas poisoning; seems well enough now." Now, if after examination of the patient there were no symptoms or signs of gas poisoning present when the patient was admitted, what practical advantage would there be in detailing the various organs and writing the word "Normal" after them? The result would be, no doubt, more pleasing to the official formalist, but no further information would be conveyed than is summed up in the words "seems well enough now." Under such circumstances, the proof that carbonic-acid poisoning existed must obviously rest with the medical man who saw the case when symptoms were present. The writer's complaint, however, is that no observations had been made on the card implying that a thorough examination had been made. The answer to this is that a medical man will not make a statement that a patient is well unless he has made an examination that will justify him in doing so. If we cannot assume this, then no system is any safeguard. It is as easy for a dishonourable medical officer to make an elaborate note as a simple note from a perfunctory examination.

The writer's remarks on *post-mortem* records are as incorrect as his remarks on the clinical notes. In all the infirmaries with which I am acquainted records of *post-mortem* examinations are made on the patient's case-paper or in a special book. In the case of autopsies performed on a Coroner's order, most Coroners provide an official paper with headings covering all the chief organs and other particulars, which has to be filled in and signed by the medical man making the *post-mortem*. In most infirmaries a copy of this official form, when filled in, is made and filed along with the patient's record-papers and charts.

But although the clinical work at the infirmaries and the notes kept of that work are far higher than is suggested by the writer of the article, it must be admitted that on the whole they fall below the level of the large voluntary hospitals. Where they fail is in the more special and delicate methods of clinical examination. Work such as blood counts, the chemical and microscopic examination of fæces, stomach contents, cerebro-spinal fluid, and similar material, bacterioscopic examinations, the microscopic examination of tumours and other similar specialised work cannot be carried out to anything like the extent it should be, because neither the strength nor the character of the staff will permit its being done. Another defect, and one shared with most of the voluntary hospitals, which have not the same excuse in the paucity of the staff, consists in the method of keeping the records. As a rule these are merely indexed under the name of the patient, with sometimes a cross-indexing for a few important sections, such as operations and infectious diseases. There is, with the exception of one or two infirmaries, no system of cross-indexing which would permit easy reference to all the cases of a given disease or group of diseases admitted to the infirmary during a given period. For a system of this kind we should have to go to America, and how far they are ahead of us in this respect may be seen in the article on "Hospital Statistics" in The Hospital of July 18, 1914.

